# Yeast β-Glucan Altered Intestinal Microbiome and Metabolome in Older Hens

**DOI:** 10.3389/fmicb.2021.766878

**Published:** 2021-12-17

**Authors:** Wenrui Zhen, Yuchen Liu, Yujing Shao, Yanbo Ma, Yuanyuan Wu, Fangshen Guo, Waseem Abbas, Yuming Guo, Zhong Wang

**Affiliations:** ^1^Henan International Joint Laboratory of Animal Welfare and Health Breeding, College of Animal Science and Technology, Henan University of Science and Technology, Luoyang, China; ^2^State Key Laboratory of Animal Nutrition, College of Animal Science and Technology, China Agricultural University, Beijing, China; ^3^National Engineering Laboratory for Animal Breeding and Key Laboratory of Animal Genetics, Breeding and Reproduction, Ministry of Agriculture and Rural Affairs, College of Animal Science and Technology, China Agricultural University, Beijing, China; ^4^College of Biology, China Agricultural University, Beijing, China

**Keywords:** yeast β-glucan, intestinal mucosal immune responses, gut microbiome, gut metabolome, hens

## Abstract

The prebiotics- and probiotics-mediated positive modulation of the gut microbiota composition is considered a useful approach to improve gut health and food safety in chickens. This study explored the effects of yeast β-glucan (YG) supplementation on intestinal microbiome and metabolites profiles as well as mucosal immunity in older hens. A total of 256 43-week-old hens were randomly assigned to two treatments, with 0 and 200 mg/kg of YG. Results revealed YG-induced downregulation of toll-like receptors (TLRs) and cytokine gene expression in the ileum without any effect on the intestinal barrier. 16S rRNA analysis claimed that YG altered α- and β-diversity and enriched the relative abundance of class *Bacilli*, orders *Lactobacillales* and *Enterobacteriales*, families *Lactobacillaceae* and *Enterobacteriaceae*, genera *Lactobacillus* and *Escherichia–Shigella*, and species *uncultured bacterium*-*Lactobacillus*. Significant downregulation of cutin and suberin, wax biosynthesis, atrazine degradation, vitamin B6 metabolism, phosphotransferase system (PTS), steroid degradation, biosynthesis of unsaturated fatty acids, aminobenzoate degradation and quorum sensing and upregulation of ascorbate and aldarate metabolism, C5-branched dibasic acid metabolism, glyoxylate and dicarboxylate metabolism, pentose and glucuronate interconversions, steroid biosynthesis, carotenoid biosynthesis, porphyrin and chlorophyll metabolism, sesquiterpenoid and triterpenoid biosynthesis, lysine degradation, and ubiquinone and other terpenoid-quinone biosyntheses were observed in YG-treated hens, as substantiated by the findings of untargeted metabolomics analysis. Overall, YG manifests prebiotic properties by altering gut microbiome and metabolite profiles and can downregulate the intestinal mucosal immune response of breeder hens.

## Introduction

The gastrointestinal tract of chickens demonstrates a highly diverse ecosystem, harboring more than 900 bacterial species (Gong et al., 2002). Accumulating studies have validated the significant role of the gut microbiota in feed digestion, nutrient absorption, breakdown of toxins, exclusion of pathogens, intestinal development, development, and stimulation of the immune system, which maintains the homeostasis of the gastrointestinal tract and endocrine activity in mammals as well as in chickens (Yeoman and White, 2014; Lynch and Pedersen, 2016; Thaiss et al., 2016). Moreover, a variety of bioactive substances such as peptidoglycans, lipopolysaccharides, DNA and extracellular vesicles, and metabolites, including short-chain fatty acids (SCFAs), aryl hydrocarbon receptor ligands, polyamines, trimethylamine, secondary bile acids, bacteriocins, quorum-sensing autoinducers, vitamins, carotenoids, neurotransmitters, and phenolic compounds, are contributed by the intestinal flora. By interacting with the host cells *via* the portal vein, these substances are directly or indirectly involved in diverse physiological processes, including host development, metabolism, cell-to-cell signal communication, immune regulation, health, and diseases (Clemente et al., 2012; Nicholson et al., 2012). Research has highlighted several predisposing factors, such as genetics, environment, age, diet, additives, antibiotics, and pathogens, that influence or regulate the host gut microbiota composition, diversity, and function, thereby altering metabolism and immunity (Belkaid and Hand, 2014; Lynch and Pedersen, 2016; Thaiss et al., 2016). An attractive approach for the amelioration of host production performance, immunity, regulation of metabolism, and prevention or treatment of diseases includes beneficial modulation of the gut microbial communities through dietary interventions or nutritional strategies (Flint et al., 2015). There is increasing evidence that polysaccharides from plants and microbes modulate the composition of the gut microbiota as well as microbial-derived metabolites in humans, animals, and poultry, which in turn improves host immunity, meaning they could be used to treat several ailments (Jayachandran et al., 2018; Tang et al., 2019; Yin et al., 2020). For example, polysaccharides extracted from purple sweet potato were found to alleviate immunosuppression by increasing the relative abundances of SCFA-producing bacteria in cyclophosphamide-treated mice (Tang et al., 2018). Metabolizing prebiotic polysaccharides, gut microbiota can produce a wide range of primary and secondary metabolites, some of which can, in turn, affect host physiology and immunity (Koh et al., 2016).

Extracted from *Saccharomyces cerevisiae* cell wall, yeast β-glucan (YG) is a kind of polysaccharide comprising a β-1,3-linked d-glucopyranosyl backbone with β-1,6-linked side chains. Numerous studies in mammals reported a range of beneficial biological activities of yeast β-glucans, including immunoregulatory, anti-inflammatory, antibacterial, antiviral, antioxidative, antitumor, antiaging, wound-healing, serum cholesterol and glucose reduction, obesity prevention, and toxin-absorption activities (Soltanian et al., 2009; Samuelsen et al., 2014). Consequently, yeast β-glucan preparations have been popularly promoted as daily nutritional and medicinal supplements in humans to enhance immunomodulatory function (Stier et al., 2014; Jayachandran et al., 2018; Wang et al., 2020b). Furthermore, as effective antibiotic alternatives, yeast β-glucan has been applied in poultry production for its beneficial growth-promoting, strong immune-regulating, anti-inflammatory, anti-infective, and immune adjuvant effects (Cox et al., 2010a; Stier et al., 2014; Vetvicka and Oliveira, 2014; Moon et al., 2016; de Vries et al., 2020). Recently, several studies identified the prebiotic properties and health-promoting benefits of yeast β-glucan, which are facilitated by modulating porcine and murine gut microbiota as well as regulating intestinal immune responses (Sweeney et al., 2012; Charlet et al., 2018; Gudi et al., 2019; de Vries et al., 2020; Wang et al., 2020a). Nonetheless, until now, few studies have evaluated the impact of feeding yeast β-glucan on the hen intestinal microbiome and metabolome.

Significant alteration in the gut microbiota in egg-laying hens is noted from the day of hatching until 60 weeks of age and was further modified as it aged (Callaway et al., 2009; Videnska et al., 2014). With increased weeks of age, hens gradually exhibit age-related deteriorations, mainly, a degenerative digestive system; compromised feed nutrient utilization; decline in productive performance, egg quality, and reproductive performance; increased intestinal permeability; chronic intestinal inflammation; reduced antioxidant capacity; and poor health status, incurring major economic loss for the poultry industry (Joyner et al., 1987; Bain et al., 2016). Significant shifts of the intestinal microbiota are suggested to be responsible for these changes. Some studies have indicated that nutritional strategies can positively regulate the gut microbiota and mucosal immunity and thus can be employed as a novel and effective approach to improving egg performance, egg quality, immunity, and antioxidant capacity in the later laying period of hens (Pan and Yu, 2014; Lee et al., 2016; Wang et al., 2018). Our previous study reported the efficacy of yeast β-glucan in enhancing the cellular and humoral immune function as well as in reducing mortality and improving egg quality and reproductive performance in aged hens (Zhen et al., 2020). Nevertheless, the impact of yeast β-glucan supplementation on both the intestinal microbiome and intestinal metabolites in older hens has rarely been investigated to date. This study aimed to explore whether yeast β-glucan can regulate the immune function of older hens by affecting gut microbiota composition and their metabolism. Thus, we determined the gut microbiome, microbial metabolite profiles, and the expression of immunity-related genes in the ileal mucosa of aged breeder hens after being fed yeast β-glucan.

## Materials and Methods

### Experimental Design, Diets, Animal Management, and Sample Collection

The China Agricultural University Animal Care and Use Committee, Beijing, China, approved the experimental animal protocols for this study (permit number SYXK 2019-0026). Two hundred and fifty-six 43-week-old Hy-Line brown breeder hens were randomly divided into two groups with eight replicates (16 hens per replicate). Hens received basal diets without or with 200 mg/kg yeast β-glucan (Glu200). The amount of yeast β-glucan supplementation was derived from our dose–response study, conducted before (Zhen et al., 2020). The yeast β-glucan used in this study was acquired from Zhuhai TianXiangYuan Biotech Holding Co., Ltd. (Zhuhai city, Guangzhou province, China) with a purity of ≥80% and molecular weight of 1,223 kDa. Its structure characterization has been described previously (Zhen et al., 2020). The composition of the corn–soybean meal-based diet used in this study followed the requirements of the National Research Council (1994). The specific composition of the basal diet and nutrient levels was the same as in our previous article (Zhen et al., 2020). Hens were reared in cages under a daily regimen of lighting (16L:8D) and were provided with feed and water *ad libitum*. Room temperature was maintained between 18 and 23°C. The study lasted 9 weeks with a 7-day adaptation period. At 51 weeks of age, one chicken was randomly chosen from each replicate and euthanized by cervical dislocation. Ileal contents and ileum were collected and frozen by liquid nitrogen, stored at −80°C until further analysis.

### Quantitative Real-Time PCR

Total RNA was extracted from snap-frozen ileal mucosa (50 mg) by the RNAiso Plus Kit (TaKaRa, Dalian, China). The purity and concentration of the total RNA were estimated by a spectrophotometer (NanoDrop 2000, Thermo Fisher Scientific, Waltham, MA, United States). cDNA was synthesized by employing a Primer Script™ RT reagent Kit with a gDNA Eraser (Takara Biotechnology Co. Ltd., Tokyo). Sangon Biotech (Shanghai) Co., Ltd., synthesized the oligonucleotide primers for chicken TLR2, TLR4, TLR6, IL-1β, IL-2, IL-6, IL-8, IL-10, IL-12, NF-κB, MyD88, IFN-γ, TGF-β3, TNF-α, CLDN1, FABP2, ZO-1, occludin, and β-actin (Table 1). Quantitative real-time PCR was executed following the protocol reported by Zhen et al. (2020). Gene expressions for TLR2, TLR4, TLR6, IL-1β, IL-2, IL-6, IL-8, IL-10, IL-12, NF-κB, MyD88, IFN-γ, TGF-β3, TNF-α, CLDN1, FABP2, ZO-1, occludin, and β-actin were analyzed using β-actin as control. Each sample gene expression was calculated by the 2^−ΔΔCt^ method (Livak and Schmittgen, 2001).

**Table 1:** Sequences of the oligonucleotide primers used for quantitative real-time PCR.^a^

Name	Primer sequence	GenBank accession number
TLR2	F: ACCTTCTGCACTCTGCCATT	NM_204278.1
	R: TGTGAATGAAGCACCGGTAA	
TLR4	F: CCACTATTCGGTTGGTGGAC	NM_001030693.1
	R: ACAGCTTCTCAGCAGGCAAT	
TLR6	F: CCAGAAGACTTGAGCGGAACACAG	NM_001081709
	R: TCTCCTCTTCGTCTGCGTCCAC	
MyD88	F: TGCAAGACCATGAAGAACGA	NM_001030962.3
	R: TCACGGCAGCAAGAGAGATT	
NF-κB	F: TGGAGAAGGCTATGCAGCTT	NM_205134.1
	R: CATCCTGGACAGCAGTGAGA	
TNF-α	F: GAGCGTTGACTTGGCTGTC	NM_204267.1
	R: AAGCAACAACCAGCTATGCAC	
IL-1β	F: TCATCTTCTACCGCCTGGAC	NM_204524.1
	R: GTAGGTGGCGATGTTGACCT	
IFN-γ	F: CTTCCTGATGGCGTGAAGA	NM_205149.1
	R: GAGGATCCACCAGCTTCTGT	
IL-2	F: GAGTGCACCCAGCAAACTCT	NM_204153.1
	R: CCGGTGTGATTTAGACCCGT	
IL-6	F: GATCCGGCAGATGGTGATAA	NM_204628.1
	R: AGGATGAGGTGCATGGTGAT	
IL-8	F: GGCTTGCTAGGGGAAATGA	NM_205498.1
	R: AGCTGACTCTGACTAGGAAACTGT	
IL-10	F: CGCTGTCACCGCTTCTTCA	NM_001004414.2
	R: TCCCGTTCTCATCCATCTTCTC	
IL-12	F: TACTTTCCTTTGCTGCCCTTCT	NM_213571.1
	R: CAGTTCCTTTCAGTTCTGTTCCCT	
TGF-β3	F: CATCGAGCTCTTCCAGATCC	NM_205454.1
	R: GACATCGAAGGACAGCCACT	
CLDN1	F: AAGTGCATGGAGGATGACCA	NM_001013611.2
	R: GCCACTCTGTTGCCATACCA	
FABP2	F: GAAGCAATGGGCGTGAATGTGATG	NM_001007923.1
	R: TTCGATGTCGATGGTACGGAAGTTG	
ZO-1	F: ACAGCTCATCACAGCCTCCT	XM_040706827.1
	R: TGAAGGGCTTACAGGAATGG	
Occludin	F: AGTTCGACACCGACCTGAAG	NM_205128.1
	R: TCCTGGTATTGAGGGCTGTC	
β-actin	F: GAGAAATTGTGCGTGACATCA	NM_205518.1
	R: CCTGAACCTCTCATTGCCA	

a Primers were designed through a primer designing tool and synthesized by Sangon Biotech (Shanghai) Co., Ltd;

F, forward; R, reverse; TLR, toll-like receptor; MyD88, myeloid differential protein 88; NF-κB, nuclear factor κB; TNF-α, tumor necrosis factor α; IL, interleukin; IFN-γ, interferon γ; TGF-β, transforming growth factor β; CLDN1, claudin 1; FABP2, fatty acid-binding protein 2; and ZO-1, zona occludens 1.

### DNA Extraction and Illumina MiSeq Sequencing

The total bacterial DNA from the ileal contents (about 100 mg each sample) was extracted by a QIAamp Fast Stool Mini Kit (Qiagen, Hilden, Germany) according to the manufacturer’s instructions and was preserved at −80°C before further analysis. The concentrations of DNA extracts were measured on a NanoDrop 2000 spectrophotometer (Thermo Scientific, MA, United States). 16S rRNA genes of 16S V3–V4 were amplified using primers F341 and R806 (F341: ACTCCTA CGGGRSGCAGCAG, R806: GGACTACVVGGGTATCTAATC). A Qiagen Gel Extraction Kit (Qiagen, Germany) was employed to purify the PCR amplification products, which were further quantified through a Qubit 2.0 fluorometer (Thermo Fisher Scientific, Waltham, United States) to pool into an even concentration. Amplicon libraries were sequenced on the Illumina MiSeq PE250 platform (Illumina, San Diego, United States).

Raw sequences were merged by FLASH, and the merged sequences were quality-filtered by Trimmomatic. Subsequently, UCHIME was adopted to remove the chimeric sequences, obtaining the effective tags, which were further analyzed by Biomarker Technologies (Beijing) Co., Ltd. Clustering of sequences at a level of 97% similarity (USEARCH, version 10.0) was followed by filtering the operational taxonomic units (OTU) by sequencing 0.005% of all sequence numbers as a threshold. Thereafter, the Greengenes database^1^ was nurtured to process the taxonomy-based analysis of the OTUs through the RDP algorithm. Analyses of OTU extraction, overlapping of OTUs, clustering, α-diversity, and β-diversity were conducted with the help of the QIIME2 (2019.4) software with Python scripts. A species diversity matrix was presented based on binary Jaccard, Bray–Curtis, and weighted and unweighted UniFrac algorithms. The linear discriminant analysis (LDA) effect size (LEfSe) was adopted to identify the differential abundance of taxa.

PICRUSt was applied to predict the function of the ileal microbiota community (Langille et al., 2013). The predictions were summarized to multiple levels, and the Statistical Analysis of Metagenomic Profile Package (STAMP) helped to compare the functional categories between the Control and Glu200 groups (Parks et al., 2014). The raw data were uploaded to the National Center for Biotechnology Information’s Sequence Read Archive database (SRA accession: PRJNA752702).

### Sample Preparation for LC-Q-TOF/MS Analysis

Thawed ileal contents (about 100 mg) of each hen were extracted with 1 ml of pre-cooled 50% methanol, vortexed for 1 min, and incubated at room temperature for 10 min; the extraction mixture was then stored at −20°C. Following centrifugation at 4,000 *g* for 20 min, the supernatants were collected and stored at −80°C for UPLC-Q-TOF/MS analysis (ultra-performance liquid chromatography (UPLC) system (SCIEX, United Kingdom) coupled to a high-resolution tandem mass spectrometer TripleTOF 5,600 plus (SCIEX, United Kingdom) in LC-Bio Technologies (Hangzhou) Co., Ltd). Meanwhile, 10 μl of each extraction mixture was combined to obtain pooled quality control (QC) samples.

### LC-Q-TOF/MS Analysis for Ileal Contents

UPLC was used for proceeding chromatographic separations according to the procedure of Zhan et al. (2020) research. Reversed-phase separation with an ACQUITY UPLC T3 column (100 mm × 2.1 mm, 1.8 μm, Waters, United Kingdom) was employed. The temperature of the column oven was maintained at 35°C. The flow rate was 0.4 ml/min, and the mobile phase comprised solvent A (water, 0.1% formic acid) and solvent B (acetonitrile, 0.1% formic acid). Gradient elution conditions were calibrated as follows: 0–0.5 min, 5% B; 0.5–7 min, 5–100% B; 7–8 min, 100% B; 8–8.1 min, 100–5% B; and 8.1–10 min, 5% B. The injection volume for each sample was 4 μl. The metabolites eluting from the column were determined by a high-resolution tandem mass spectrometer TripleTOF 5,600 plus (SCIEX, United Kingdom). The Q-TOF was operated in both positive and negative ion modes. The curtain gas was set at 30 PSI, ion source gas 1 at 60 PSI, and ion source gas 2 at 60 PSI, and the interface heater temperature was 650°C. For positive and negative ion modes, the ionspray voltage floating was set at 5,000 and −4,500 V, respectively. The mass spectrometry data were acquired in IDA mode. The TOF mass ranged from 60 to 1,200 Da. The survey scans were acquired in 150 ms, and as many as 12 product ion scans were collected on exceeding a threshold of 100 counts per second (counts/s) and with a 1+ charge state. The total cycle time was fixed to 0.56 s. Four time bins were summed for each scan at a pulse frequency value of 11 kHz through monitoring of the 40 GHz multichannel TDC detector with four-anode/channel detection. Dynamic exclusion was set for 4 s. The mass accuracy was calibrated every 20 samples during the acquisition. Furthermore, the stability of the LC–MS during the whole acquisition was assessed by acquiring a QC after every 10 samples.

### LC-Q-TOF/MS Data Acquisition and Processing

LC–MS raw data were processed by the XCMS, CAMERA, and metaX toolbox implemented with the R software. The online KEGG and HMDB databases and an in-house fragment spectrum library of metabolites were adopted to annotate the metabolites by matching the exact molecular mass data (m/z) of samples with those from the database. The KEGG databases^2^ were used to analyze the metabolic pathways of differential metabolites. To process the intensity of peak data, metaX was used. Differences in metabolite concentrations between two treatments were identified using Student *t* tests. An FDR (Benjamini–Hochberg) and supervised partial least squares discriminant analysis (PLS-DA) were conducted to adjust the value of *p* for multiple tests and discriminate the different variables between groups through metaX, respectively. The data supporting the findings of this study have been deposited into the CNGB Sequence Archive (CNSA) of China National GeneBank DataBase (CNGBdb) with accession number CNP 0002101.

### Statistical Analysis

Immunological parameter data were analyzed using a one-way analysis of the variance procedure of SPSS 17.0 (SPSS Inc., Chicago, IL, United States). A value of *p* ≤ 0.05 was considered significant, and value of *p* at 0.05–0.10 were classified as trends.

## Results

### Gene Expression in the Ileum of Hens

As evident from Table 2, compared to the control group, significant downregulation (*p* < 0.05) of the mRNA levels of TLR2, TLR4, IL-6, IL-8, IL-10, IL-12, TGF-β3, IFN-γ, and TNF-α genes was observed in hens fed with 200 mg/kg yeast β-glucan, whereas no significant effect was documented on CLDN1, FABP2, ZO-1, occludin, TLR6, IL-1β, IL-2, MyD88, and NF-κB mRNA expression (*p* > 0.05). In general, the addition of 200 mg/kg yeast β-glucan manifested lower levels of mRNA expression of the genes in the ileum of hens as compared to the control group. However, it failed to affect the intestinal barrier.

**Table 2:** Effect of dietary yeast β-glucan supplementation on ileal mucosa gene expression in laying hens (*n* = 8).

Items	Control	Yeast β-glucan	SEM^a^	*p*
TLR2	1.00	0.80	0.046	0.021
TLR4	1.00	0.72	0.055	0.004
TLR6	1.00	1.09	0.074	0.569
MyD88	1.00	0.85	0.059	0.207
NF-κB	1.00	0.93	0.061	0.601
IL-1β	1.00	0.90	0.052	0.352
IL-2	1.00	0.99	0.076	0.964
IL-6	1.00	0.63	0.093	0.039
IL-8	1.00	0.51	0.086	0.000
IL-10	1.00	0.80	0.053	0.048
IL-12	1.00	0.69	0.077	0.039
TGF-β3	1.00	0.83	0.037	0.013
IFN-γ	1.00	0.54	0.111	0.030
TNF-α	1.00	0.76	0.050	0.008
CLDN1	0.89	0.75	0.090	0.462
FABP2	0.64	0.70	0.155	0.859
ZO-1	1.00	1.00	0.076	0.979
Occludin	1.00	1.29	0.120	0.240

a SEM, standard error of the mean.

### The Diversity and Composition of Gut Microbiota

**Figure 1:**
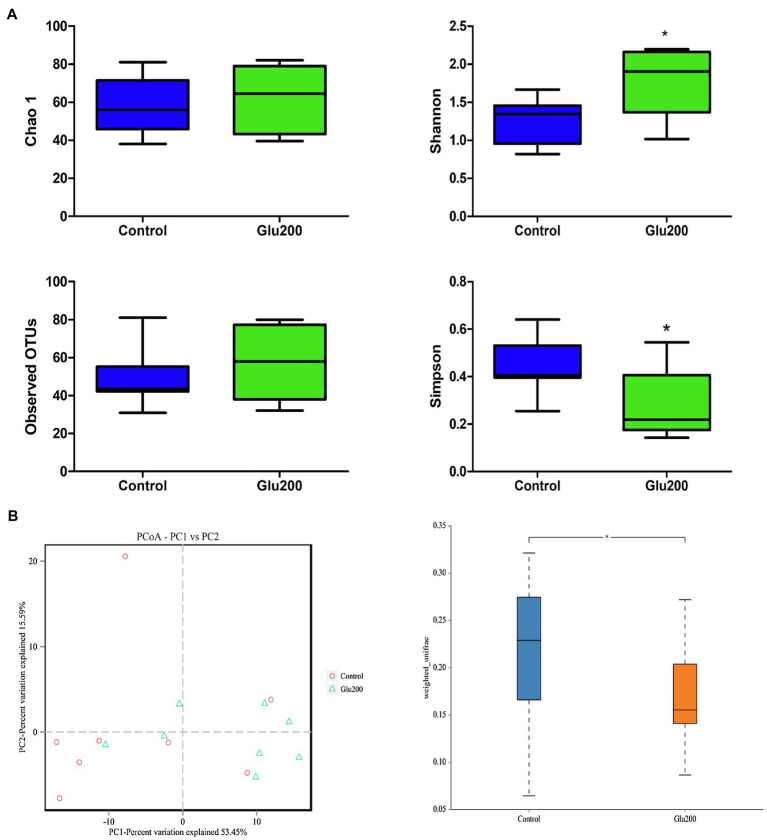
Differences in bacterial community diversity, richness, and structures in the ileum of breeder laying hens fed with or without dietary Glu200. **(A)** Community diversity and richness between Control and Glu200 groups. **(B)** PCoA of bacterial community structure between Control and Glu200 groups. Each symbol represents each gut microbiota. Red symbols represent the Control group, and blue symbols represent the Glu200 group. Control: the basal diet; Glu200: the basal diet supplemented with 200 mg/kg yeast β-glucan. *means significant difference between groups.

16S rRNA gene sequencing facilitated an average of 69,219 high-quality sequences per sample for the following analysis. Based on the 97% sequence similarity, these sequences were distributed to 93 OTUs. α-Diversity is illustrated in Figure 1A, and yeast β-glucan supplementation significantly enhanced the Shannon index, while reducing the Simpson index, relative to the control group (*p* < 0.05). The results of β-diversity [principal components analysis (PCA) and principal coordinates analysis (PCoA)] reflected a notable difference in gut microbiota structure and composition between the two groups (Figure 1B).

**Figure 2:**
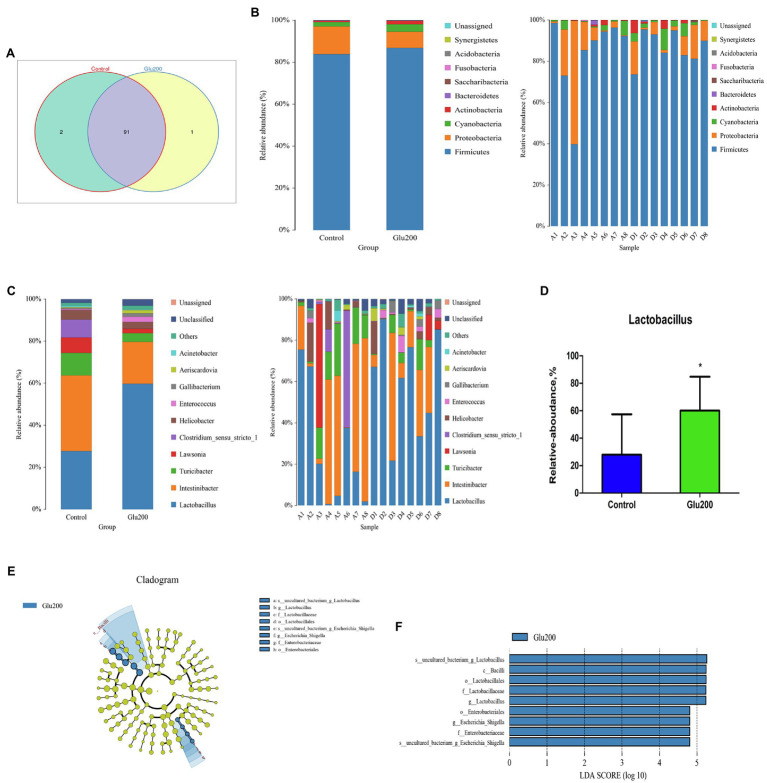
Changes of microbial composition in the ileum of breeder laying hens fed with or without dietary Glu200. **(A)** Venn diagram for bacterial OTU compositions in two groups. **(B)** Microbial composition at the phylum level. **(C)** Microbial composition at the genus level; each bar represented the average relative abundance of each bacterial taxon within a group. **(D)** Difference of the relative abundances of *Lactobacillus* between Control and Glu200 groups. LEfSe was used to explore differences between Control and Glu200 groups. **(E)** Cladogram plot of LEfSe analysis. **(F)** Histogram of LDA value distribution between Control and Glu200 groups. *means significant difference between groups.

A Venn diagram identified two unique OTUs in the control group and one unique OTU in the Glu200 group (Figure 2A). Ileal microbiota confirmed two dominant bacteria – *Firmicutes* and *Proteobacteria* – at the phylum level (Figure 2B). Bacterial phyla portrayed no difference between the two groups (*p* > 0.05). At the genus level (Figure 2C), yeast β-glucan addition significantly increased the relative abundance of *Lactobacillus* in the ileum as compared to the control group (*p* < 0.05; Figure 2D). LEfSe analysis claimed a significantly higher relative abundance of *Lactobacillus*, *Bacilli*, *Lactobacillales*, *Lactobacillaceae*, *Lactobacillus*, *Enterobacteriales*, *Enterobacteriaceae*, and *uncultured bacterium*-*Escherichia*–*Shigella* in the Glu200 group than that in the control group (LDA score >4; Figures 2E,F).

### Predicted Function of Intestinal Microbiota

**Figure 3:**
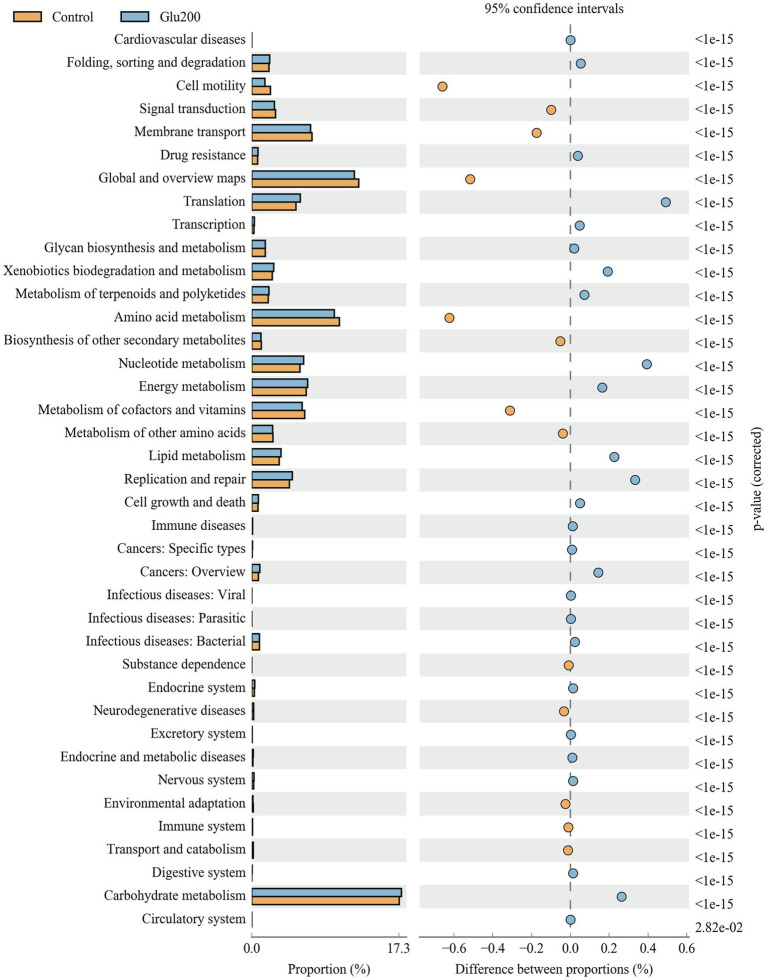
Microbial function prediction in the ileum of breeder laying hens fed with or without dietary Glu200. The second level of the KEGG pathway is shown in the extended error bar. The value of *p* are shown to the right. Control: the basal diet; Glu200: the basal diet supplemented with 200 mg/kg yeast β-glucan.

Figure 3 summarizes the microbial function prediction at level 2 of the KEGG pathways. Between the control and Glu200 groups, 38 functional pathways were identified. Compared with the control group, the yeast β-glucan group showed significant enrichment of several functional pathways, including translation, nucleotide metabolism, replication and repair, lipid metabolism, carbohydrate metabolism, xenobiotic biodegradation and metabolism, energy metabolism, metabolism of terpenoids and polyketides, folding, sorting and degradation, cell growth and death, transcription, and drug resistance, while showing suppression of other pathways such as amino acid metabolism, cell motility, metabolism of cofactors and vitamins, membrane transport, signal transduction, biosynthesis of other secondary metabolites, and metabolism of other amino acids.

### Ileal Metabolites and Metabolic Pathways

**Figure 4:**
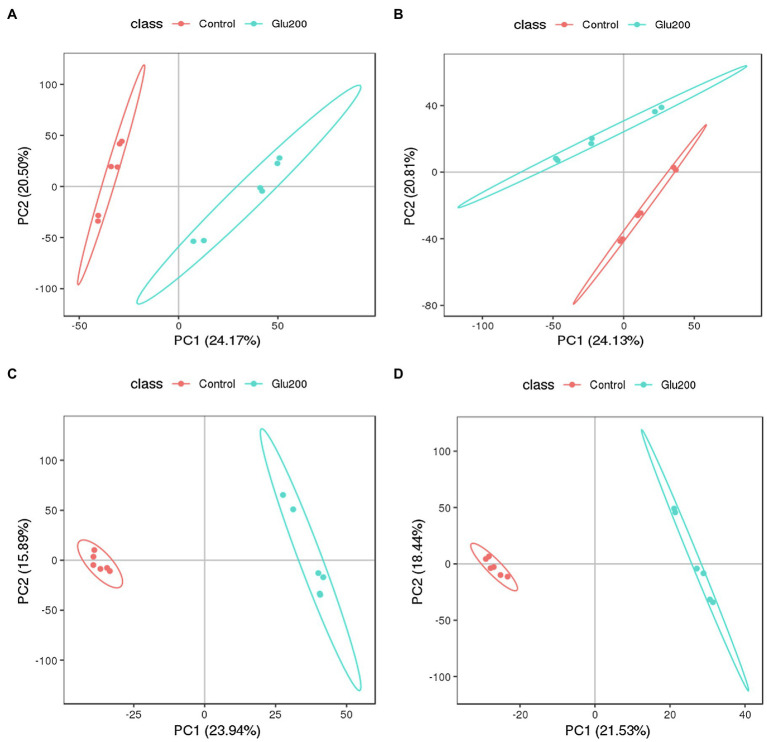
Principal components analysis (PCA) and partial least squares discriminant analysis (PLS-DA) score plots of identified metabolites in the ileum contents of chickens (*n* = 6) fed an un-supplemented control diet (Control) (red) or a basal diet supplemented with yeast β-glucan (Glu200) (blue). PCA and PLS-DA models demonstrating a separation between Control and Glu200 groups. Each dot on the plot represents the scores of the biological replicates. **(A)** PCA-positive ion; **(B)** PCA-negative ion; **(C)** PLS-DA-positive ion (R2 = 0.998, Q2 = 0.883); **(D)** PLS-DA-negative ion (R2 = 0.999, Q2 = 0.816).

To elucidate whether supplementation of yeast β-glucan affects intestinal microbiota and mucosal immune response by altering intestinal metabolites, we further estimated the intestinal metabolite profiles of the ileum contents using UPLC-MS/MS-based non-targeted metabolomics approach. The PCA and PLS-DA score plot clearly indicated a distinct separation of identified intestinal metabolites, including positive ion modes and negative ion modes between the un-supplemented control and the Glu200-supplemented groups (Figure 4). This finding confirmed the induction of significant alterations of the intestinal metabolic profiles in the hens receiving yeast β-glucan. The PLS-DA models were authenticated by cross-validation (R2 = 0.998 and Q2 = 0.883 for positive ion modes and R2 = 0.999 and Q2 = 0.816 for negative ion modes).

**Figure 5:**
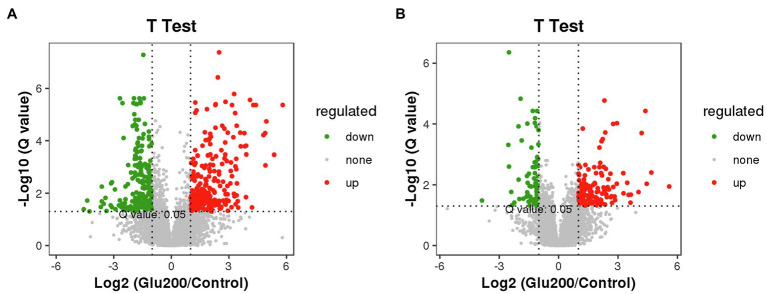
Volcano plot of differential metabolites between the control and the Glu200 groups. The abscissa axis represents the multiple of difference of metabolites (log2), and the vertical axis represents the *q*-value (−log10). Each point represents a kind of metabolite; the scatter color represents the final screening result. Significant upregulated metabolites are indicated in red (up), downregulated metabolites are indicated in green (down), and nonsignificant differences in metabolites are gray (none). **(A)** Represents positive ion modes and **(B)** represents negative ion modes.

Figure 5 highlights the variety of positive ion modes in the ileal contents, with 264 increased and 234 decreased molecular features based on a VIP >1.0 in 95% jack-knifed confidence intervals. Furthermore, the levels of 136 differential metabolites of negative ions were increased, and those of 73 negative ions were decreased.

**Figure 6:**
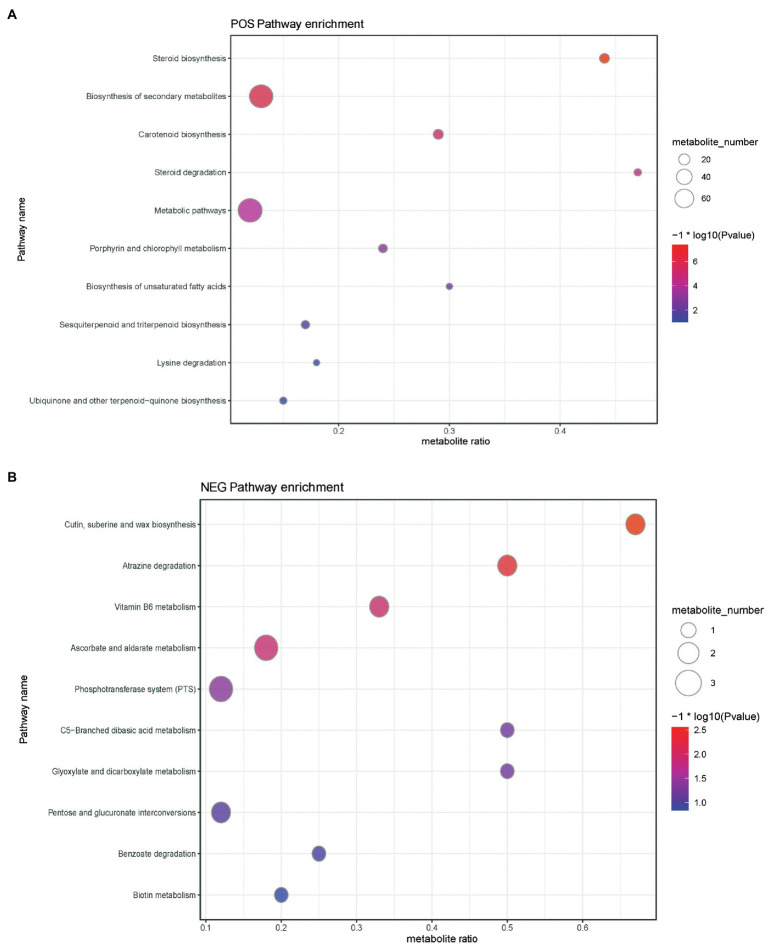
Buddle diagram of KEGG pathway enrichment analysis of differential metabolites. The *x* axis (Rich Factor) represents the ratio of the number of differential metabolites annotated to the pathway to all the metabolites annotated to the pathway. The higher the value, the higher the degree of enrichment of differential metabolites in this pathway. The color of the point represents the value of *p* value of the hypergeometric test, and the smaller the value is, the greater the reliability of the test and the more statistically significant it is. The size of the dots represents the number of differential metabolites annotated to the pathway, and the larger the point, the more differential metabolites in the pathway. **(A)** Positive ion pattern analysis and **(B)** negative ion pattern analysis.

Metabolic pathways presenting with a difference are detailed in Figure 6 and Supplementary Tables S1–S4. Results disclosed that the levels of differential metabolites related to cutin, suberin, wax biosynthesis, atrazine degradation, vitamin B6 metabolism, phosphotransferase system (PTS), steroid degradation, biosynthesis of secondary metabolites, biosynthesis of unsaturated fatty acids, aminobenzoate degradation, and quorum sensing were significantly downregulated (Supplementary Tables S1 and S2), whereas the levels of differential metabolites mapped to ascorbate and aldarate metabolism, C5-branched dibasic acid metabolism, pentose and glucuronate interconversions, glyoxylate and dicarboxylate metabolism, benzoate degradation, ubiquinone and other terpenoid-quinone biosyntheses, steroid biosynthesis, carotenoid biosynthesis, porphyrin and chlorophyll metabolism, metabolic pathways, sesquiterpenoid and triterpenoid biosyntheses, and lysine degradation were upregulated (Supplementary Tables S3 and S4) in the ileal content of hens belonging to the yeast β-glucan supplementation group compared with the non-supplemented control group.

## Discussion

The rapid evolution of research in the field of prebiotics and immunostimulants is attributed to this sustainable method to improve chicken health that is subjected to fewer regulatory restrictions on food safety than other methods (Lillehoj and Lee, 2012). Because of their strong immunomodulatory potential, yeast β-glucan, a polysaccharide, finds significant application as a feed supplement for the poultry industry (Soltanian et al., 2009; McFarlin et al., 2013; Samuelsen et al., 2014; Stier et al., 2014; Fuller et al., 2017; Jayachandran et al., 2018). Our previous study indicated that dietary yeast β-glucan induced significant escalation of systemic immunity and reduction of the mortality rate of laying hens and improved egg quality and fertile egg hatchability, suggesting beneficial effects of yeast β-glucan addition on the reproductive performance of aged hens (Zhen et al., 2020). Gut health exerts important effects on egg quality and reproductive performance. To justify the cause of yeast β-glucan treatment-mediated beneficial effects on egg yolk color and fertile egg hatchability, we first investigated the impact of yeast β-glucan treatment on gene expression of intestinal mucosal immune-related factors. Interestingly, our observation confirmed the downregulation of ileal mucosal TLR-mediated immune responses on feeding 200 mg/kg yeast β-glucan. Immunity and inflammation in the gut are significantly monitored by the TLR-mediated signal pathway and its related effector molecules (Abreu, 2010; Shao et al., 2016; Nawab et al., 2019). Declined TLR-mediated immune-related genes expressions were observed in the ileum of aged hens following yeast β-glucan treatment, suggesting that, during the later laying period of hens, the inclusion of yeast β-glucan into the diet could alleviate intestinal chronic inflammatory responses resulting from age-related gut dysbiosis, thereby improving immune status. In line with our findings, previous researches have substantiated the role of supplementation of yeast β-glucan in ensuring homeostasis of the organism and in protecting chicken against an excessive immune response when subjected to pathogen challenge or other inflammatory stimuli by inhibiting the activation of the TLR-mediated signal pathway and restoring the balance of immune responses (Tzianabos, 2000; Huff et al., 2006; Chen et al., 2008; Cox et al., 2010a,b; Jawhara et al., 2012; Shao et al., 2013, 2016; Tian et al., 2016). Therefore, we proposed that yeast β-glucan supplementation downregulated ileal immune responses, which might be beneficial to gut health and the intestinal integrity of older hens. Tight junction proteins CLDN1, ZO-1, and occludin along with FABP2 are used as biomarkers to predict gut barrier function and health in chicken (Chen et al., 2015). Nonsignificant changes in CLDN1, FABP2, ZO-1, and occludin mRNA levels following yeast β-glucan administration in our study suggested that feeding yeast β-glucan to older hens failed to impair intestinal barrier function and integrity. The above observations indicated that the improvement in egg performance and egg quality by yeast-derived β-glucan might be attributed to reducing the intestinal chronic inflammatory state caused by aging, maintaining gut homeostasis, and ameliorating gut function in aged hens.

Recently, growing evidence has substantiated the association of gut microbiota together with their metabolites to the immune regulation indulged in local and systemic inflammatory responses (Abreu, 2010; Belkaid and Hand, 2014; Bae et al., 2020; Lavelle and Sokol, 2020). To further elucidate the mechanism of inhibition of intestinal immune responses in aged hens mediated by yeast β-glucan treatment, the modulatory effect of yeast β-glucan administration on gut microbiome and metabolome was evaluated. In this study, increased Shannon index, decreased Simpson index, and altered ileal microbiota community composition and structure indicated that yeast β-glucan supplementation into the diet significantly affected the α-diversity and β-diversity of the ileal microbiota. A more diverse microbial community signified the stronger homeostasis of the gut microbial community. Significant alteration of the yeast β-glucan treatment-induced ileal microbiota α-diversity and β-diversity of aged hens presumed that yeast β-glucan could ameliorate the intestinal microbial community and structure, which may be related to gut homeostasis. Interestingly, taxon analysis discovered an enriched population of ileal *Lactobacillus* in the Glu200 group relative to the control group. LEfSe analysis also confirmed enrichment of the relative abundance of *Uncultured bacterium-g-Lactobacillus*, *Bacilli*, *Lactobacillales*, *Lactobacillaceae*, *Lactobacillus*, *Enterobacteriales*, *Enterobacteriaceae*, and *uncultured bacterium-Escherichia*–*Shigella* following yeast β-glucan addition. In accordance with our observations, several recent studies using mammals as models and *in vitro* simulated fermentation systems have demonstrated yeast β-glucan-facilitated changes in the structure of gut microbiota and exhibited prebiotic-like activity. For example, Xu et al. (2020) reported that yeast β-glucan promoted alleviation of Aβ1–42-induced cognitive deficits by enriching the beneficial bacteria (*Lactobacillus* and *Bifidobacterium*) and reducing the pathogenic bacteria (*Oscillibacter*, *Mucispirillum*, *Alistipes*, *Anaerotruncus*, and *Rikenella*). Chen et al. (2019) reported improved intestinal integrity and motility function in mice with loperamide-induced constipation, induced by bread yeast β-glucans *via* the enhanced abundance of *Lactobacillus intestinalis*, *Mucispirillum*, and *Peptococcaceae*. Increased *Lactobacillus* and *Bifidobacterium* populations in the gut of the cyclophosphamide-induced chickens were attributed to sulfated yeast β-glucan, which could also effectively alleviate immunosuppression (Wang et al., 2019). Similarly, attenuation of DSS-induced colitis resulted from oral administration of soluble yeast β-glucans to mice *via* an increased population of intestinal *Lactobacillus johnsonii* and *Bacteroides thetaiotaomicron* (Charlet et al., 2018). Cao et al. (2016, 2018) also observed that enriched gut microbiota proportion of *Akkermansia* by orally administered yeast β-1,3-glucan could suppress chronic inflammation in diabetic mice. In mouse T1D (type 1 diabetes) and colitis models, supplementation with yeast β-glucans manifested anti-diabetes and anti-inflammatory effects by enriching *Bacteroidetes* and *Verrucomicrobia* while diminishing the phylum *Firmicutes* (Gudi et al., 2019, 2020). In early life, the addition of yeast β-glucans alters gut microbiota composition such as *Methanobrevibacter*, *Fusobacterium*, and *Romboutsia*, during the pre-weaning period in piglets (de Vries et al., 2020). Additionally, using a simulated large-intestine fermentation system, Wang et al. (2020a) observed that yeast β-glucan could be broken down following gut microbiota fermentation and selectively enhanced the growth of beneficial gut microbiota *Bifidobacterium longum*, impeded the proliferation of harmful intestinal flora, and decreased the ratio of *Firmicutes* to *Bacteroidetes*. Thus, our findings established that yeast β-glucan could be fermented by gut microbiota of hens, possessing prebiotic-like activity for the modulation of gut microbiota in laying hens. As an important probiotic, *Lactobacillus* stimulates the gut health of poultry (Tsai et al., 2012; Ashraf and Shah, 2014). As compared to that in the control hens, a higher proportion of *Lactobacillales*, *Lactobacillaceae*, *Lactobacillus*, and *Uncultured bacterium-g-lactobacillus* was observed in the gut of aged hens with dietary yeast β-glucan in this study, suggesting the amelioration of the balance of gut microbiota and gut health of aged hens by yeast β-glucan addition. After feeding with yeast β-glucan, intestinal immune responses declined, which was possibly related to promoting intestinal beneficial microbiota *Lactobacillus* growth. Concurrently, a remarkable escalation in the relative abundance of *Enterobacteriales*, *Enterobacteriaceae*, and *uncultured bacterium Escherichia–Shigella* was witnessed in the yeast β-glucan group compared with the blank group. Though some *Enterobacteriaceae* belonging to the *Proteobacteria* phylum are regarded as pathogens (such as *Salmonella* and *Shigella*), most *Enterobacteriaceae* in the gut is considered commensals as they benefit their host by fermenting glucose with acid production, or generating vitamin B12 and vitamin K, or promoting the maturation of the host immune system and competitively eliminating pathogens in the intestine (Leimbach et al., 2013; Conway and Cohen, 2015; Litvak et al., 2019). Considering the constructive effects of yeast β-glucan on chickens’ gut microbiota composition, the present results indicate that yeast β-glucan could improve gut health and is one of the candidate prebiotics stimulating the intestinal health of aged hens. However, further exploration is essential to elucidate the mechanism of yeast β-glucan in promoting the growth of intestinal commensal *Enterobacteriaceae*.

PICRUSt analysis determined that yeast β-glucan contributed to the enrichment of nucleotide metabolism, lipid metabolism, xenobiotic biodegradation and metabolism, carbohydrate metabolism, and energy metabolism while suppressing amino acid metabolism and metabolism of cofactors and vitamins in the ileal microbiota. Consistent with our findings, Taylor et al. (2020) reported a notable change in the function of the gut microbiota of DSS-induced mice with respect to enhanced carbohydrate metabolism, glycan biosynthesis, and metabolism, as well as fatty acid biosynthesis, resulting from β-1,3-d-glucan treatment. Wang et al. (2020a) employed a simulated large-intestine fermentation system and observed that cell motility, lipid metabolism, transport, and catabolism, transcription, enzyme families, membrane transport, neurodegenerative diseases, cellular processes, and signaling were significantly enriched in the yeast β-glucan group with respect to the control group. Similarly, yeast β-glucan supplementation indulged a shift in gut microbiota composition and structure and increased concentrations of fecal SCFAs such as acetic, propionic, and butyric acids in mice (Gudi et al., 2020) and Alzheimer’s disease-induced mice (Xu et al., 2020). As reported, gut microflora could metabolize nondigestible carbohydrates into SCFAs, which are known to boost intestine health by its trophic, anti-inflammatory, and immunomodulatory effects (Lavelle and Sokol, 2020). Hindgut microbiota usually ferments amino acids into amines, ammonia, and gases such as sulfide, methane, and hydrogen, which are genotoxins, cytotoxins, and carcinogens associated with colon cancer and inflammatory bowel diseases (Neis et al., 2015; Lavelle and Sokol, 2020). Although there were varied reports regarding gut microbiota composition and structure, along with predicted microbial community functions following yeast β-glucan treatment in different experimental studies, based on our observation, we opined that dietary yeast β-glucan administration is beneficial in modifying the gut microbiota in composition and community structure toward enhanced carbohydrate metabolism and suppressed amino acid metabolism, thereby contributing to dampening of age-related gut chronic inflammation in hens. The inhibition of TLR-NF-κB signaling pathway and reduced intestinal pro-inflammatory IFN-γ, TNF-a, and IL-8 levels upon yeast β-glucan treatment in our study lends support regarding this notion. Therefore, 16S gene sequence analysis emphasized the prebiotics-like effect of yeast β-glucan in hens that could amend their gut health.

The gut microbiome interferes with intestinal functions through its metabolites. In this study, UPLC-MS/MS-based nontargeted metabolomics analysis revealed that yeast β-glucan supplementation significantly downregulated the levels of differential metabolites related to the PTS, quorum sensing, biosynthesis of unsaturated fatty acid, steroid degradation, and aminobenzoate degradation pathway, compared with the control (Murakami et al., 1993; Liu et al., 2013; Zhao et al., 2019). The involvement of differential metabolites (*N*-acetyl-d-glucosamine (GlcNAc) and *N*-acetyl-d-galactosamine) related to the PTS pathway was noted in regulating the virulence of certain pathogens and anti-inflammatory effects. Antioxidant, antiviral, and anti-inflammatory activities were contributed by several metabolites (13,16-docosadienoic acid, erucic acid, eicosadienoic acid, and 5Z,8Z,11Z,14Z,17Z-eicosapentaenoic acid) engaged in the biosynthesis of the unsaturated fatty acid pathway (Weldon et al., 2007; Chen et al., 2020; Liang et al., 2020). Immunosuppressive effects were manifested by the metabolites (androstenedione, 5-androstene-3, 17-dione, testosterone, 5-α-androstane-3, 17-dione, dehydroepiandrosterone, and 3,4-dihydroxy-9,10-secoandrosta-1,3,5(10)-triene-9,17-dione) pertaining to steroid degradation (Muriel et al., 2017). The adhesion of flagellum, biofilm formation, virulence factor gene transcription, and production of toxins of bacteria are affected by bacterial pheromones (*N*-hexanoyl-l-homoserine lactone, *N*-heptanoyl-homoserine lactone, and *N*-octanoyl-l-homoserine lactone) involved in the quorum-sensing pathway, thereby directly promoting the infection by pathogens (Yang et al., 2013; Li et al., 2018). Differential metabolites (anthranilate, 4-aminobenzoate, (*S*)-4-hydroxymandelate, vanillate, and 3-hydroxy-5-oxohexanoate) mapped to aminobenzoate degradation have been reported to interfere with bacterial biofilm formation and, at the same time, exhibit immunosuppressive properties for inflammation-related diseases without inducing cell death (Li et al., 2017). Thus, reduced differential metabolites and related metabolic pathways in the ileal content of hens following yeast β-glucan administration indicated yeast β-glucan-mediated inhibition or regulation of the growth and the virulence factor production of intestinal potential pathogens and prevention of intestinal pathogens’ infection, thereby suppressing intestinal inflammatory responses of older breeding hens or maintaining gut immune homeostasis. The decline in the intestinal pheromone homoserine lactone content, which was also involved in the virulence factor gene transcription of intestinal potential pathogens and increase in the relative abundance of lactobacillus, after feeding with yeast β-glucan, further confirmed this observation.

Downregulation of the vitamin B6 metabolism pathway following yeast β-glucan treatment in the present study was also consistent with the outcome of the PICRUSt prediction from this study. Vitamin B6 has been known to participate in all amino acid metabolism, mainly in the form of pyridoxal 5-phosphate as a coenzyme in the transamination reaction (Wilson et al., 2019). This observation may imply that feeding yeast β-glucan could impede the amino acid utilization by ileal microbiota or reduce amino acid deamination in the hindgut *via* suppressing the vitamin B6 metabolism pathway of hindgut microbiota.

On the other hand, our research highlighted downregulation in the levels of different metabolites involved in cutin, suberin, and wax biosynthesis pathway, after feeding with yeast β-glucan, while it highlighted upregulation in the ascorbate and aldarate metabolism, C5-branched dibasic acid metabolism, pentose and glucuronate interconversions, glyoxylate and dicarboxylate metabolism, steroid biosynthesis, porphyrin and chlorophyll metabolism, carotenoid biosynthesis, ubiquinone, and other terpenoid-quinone biosyntheses, sesquiterpenoid and triterpenoid biosyntheses, and lysine degradation, in the ileal content of hens belonging to the yeast β-glucan supplementation group compared with those belonging to the non-supplemented group. Differential metabolites (cis-9,10-epoxystearic acid, 18-hydroxyoleate) related with cutin, suberin, and wax biosynthesis pathway were responsible for the lipid metabolism disorder of cells by increasing the intracellular lipid content, as well as inhibiting the fatty acid oxidation in peroxisomes and mitochondria (Liu et al., 2020). Antioxidative, antiaging, anti-inflammatory, and immunomodulatory effects, as well as improvement in lipid metabolism, were attributed to the biomarkers (α-tocotrienol, δ-tocopherol, 2-methyl-6-phytylquinol, γ-tocopherol, β-tocopherol, and 2,3-dimethyl-5-phytylquinol) engaged in ubiquinone and other terpenoid-quinone biosynthesis pathways (Jiang, 2014; Galli et al., 2017). Differential metabolites (ascorbate, d-glucuronolactone, and 5-dehydro-4-deoxy-d-glucuronate) which participated in ascorbate and aldarate metabolism and pentose and glucuronate interconversions manifested various functions, such as antioxidant, immunoregulatory, and/or liver-detoxification function (Zhu et al., 2019; Gouda et al., 2020). Anti-oxidative and anti-inflammatory potential of differential metabolites (zeaxanthin, adonixanthin, and lutein) mapped to carotenoid biosynthesis encouraged their use as a feed additive for the coloring of poultry meat and eggs (Rao and Rao, 2007; Maoka et al., 2013; Moraes et al., 2016; Milani et al., 2017). Association of biomarkers (farnesol and nerolidol) mapped to sesquiterpenoid and triterpenoid biosynthesis with antioxidative and anti-inflammatory properties was documented (Jahangir et al., 2006; Khan and Sultana, 2011). Similarly, *in vivo* and *in vitro* studies have reported that yeast β-glucan portrayed antioxidative and anti-inflammatory activities in mice (Lei et al., 2015; Cao et al., 2016, 2018; Charlet et al., 2018; Gudi et al., 2019, 2020). Moreover, supplementation of tocopherol, lutein, or ascorbate was found to increase the fertility and hatchability of breeding eggs in poultry (Elibol et al., 2001; Panda et al., 2008; Zhu et al., 2019). Furthermore, our results also claimed enrichment of porphyrin and chlorophyll metabolism pathways in the ileal contents of hens receiving yeast β-glucan. The main eggshell pigment in brown-shelled eggs was identified to be the differential metabolite-protoporphyrin involved in porphyrin and chlorophyll metabolism pathways (Li et al., 2013). Hence, increase in these differential metabolites after yeast β-glucan addition indicated that yeast β-glucan might possess antioxidative, anti-inflammatory, antiaging, and liver-detoxification functions along with modulating lipid metabolism and pigment formation of brown-shelled eggs through modification of the above metabolic pathways, resulting in improved egg color, enhanced fertile egg hatchability of aged hens, and decreased intestinal immune-inflammatory responses after feeding yeast β-glucan.

Interestingly, significant amplification in the concentration of calcidiol, 7-dehydrocholesterol, vitamin D3, and cholesterol involved in the steroid biosynthesis pathway was noted in the ileal content of the yeast β-glucan group compared with the control group. The breeder hens’ absorption of calcium and phosphorus was augmented by calcidiol and vitamin D3, thereby promoting growth bone and improving eggshell quality and fertile egg hatchability (Wen et al., 2019; Adhikari et al., 2020). Recently, the most striking observation is that vitamin D3 and its derivative supplementation were reported to not only display immunomodulatory and anti-inflammatory properties but could also stimulate the growth of gut beneficial microbiota as well as reinforce intestinal barrier function, thereby enhancing host disease resistance (Sassi et al., 2018; Fakhoury et al., 2020). Thus, overrepresented differential metabolites mapped to steroid biosynthesis pathway after yeast β-glucan administration provide compelling evidence for yeast β-glucan-mediated suppression of intestinal inflammation and improved egg quality and reproductive performance of hens in the later laying period. Collectively, the results of metabolomics analysis claimed that yeast β-glucan might possess new functions, including anti-oxidative, liver-detoxification, as well as modulation of lipid metabolism through altered gut microbiota metabolic pathway, apart from immunomodulatory effects in hens.

Future research will need to further confirm these observations and investigate the underlying mechanism of yeast β-glucan in improving gut health in more depth by employing different experimental models. Furthermore, the correlation between gut microbiota, gut microbiome, and intestinal mucosal immune responses also needs to be further explored.

## Conclusion

The present study initially reported the prebiotic-like properties manifested by dietary yeast β-glucan provided to aged hens by altering gut microbiome and metabolite profiles of the ileal content. Furthermore, dietary yeast β-glucan supplementation could repress the ileal chronic inflammation of breeder hens in the later laying period. Overall, this study sheds light on a promising strategy for the prevention of age-related immune hypofunction or chronic intestinal inflammation in aged hens with the help of dietary-supplement-based immunomodulators.

## Data Availability Statement

The datasets presented in this study can be found in online repositories. The names of the repository/repositories and accession number(s) can be found at: https://www.ncbi.nlm.nih.gov/, PRJNA752702; https://db.cngb.org/, CNP 0002101.

## Ethics Statement

The animal study was reviewed and approved by China Agricultural University Animal Care and Use Committee.

## Author Contributions

ZW and WZ designed the research. WZ performed the research and wrote the manuscript. WZ and YL analyzed the data. ZW, YS, YM, YW, FG, WA, and YG participated in the revision of the manuscript. All authors contributed to the data interpretation and approved the final version of the manuscript.

## Funding

This research was funded by Zhuhai TianXiangYuan Biotech Holding Co., Ltd. Funders had no role in the study design, analysis, or writing of this article.

## Conflict of Interest

The authors declare that the research was conducted in the absence of any commercial or financial relationships that could be construed as a potential conflict of interest.

## Publisher’s Note

All claims expressed in this article are solely those of the authors and do not necessarily represent those of their affiliated organizations, or those of the publisher, the editors and the reviewers. Any product that may be evaluated in this article, or claim that may be made by its manufacturer, is not guaranteed or endorsed by the publisher.

## Footnotes

^1^ http://greengenes.lbl.gov

^2^ http://www.genome.jp/kegg/
