# Supplementary material for: Yeast β-Glucan Altered Intestinal Microbiome and Metabolome in Older Hens
Source: Front Microbiol. 2021 Dec 17;12:766878. doi: 10.3389/fmicb.2021.766878 (PMC8718749; doi:10.3389/fmicb.2021.766878)
Supplement: Supplementary file 1 [file Table_1.DOCX]

**TABLE S1.** Downregulated metabolites negative ion involved pathways in yeast β-glucan supplementation group compared with control group.

| Pathway | Different metabolites | The number of different metabolites^a^ | The number of all metabolites^b^ | P value^c^ |
| --- | --- | --- | --- | --- |
| Cutin, suberine and wax biosynthesis | cis-9,10-Epoxystearic acid, 18-Hydroxyoleate | 2 | 3 | 0 |
| Atrazine degradation | N-Cyclopropylammelide, N-Cyclopropylammeline | 2 | 4 | 0.001 |
| Vitamin B6 metabolism | 4-Pyridoxate, 3-Hydroxy-4-hydroxymethyl-2-methylpyridine-5-carboxylate | 2 | 6 | 0.002 |
| Phosphotransferase system (PTS) | N-Acetyl-D-glucosamine, N-Acetyl-D-galactosamine | 2 | 26 | 0.029 |

^a^ The number of differential metabolites that hit the pathway.

^b^ The number of metabolites in the pathway.

^c^ The P value of metabolic pathway enrichment analysis.

**TABLE S2.** Downregulated metabolites positive ion involved pathways in yeast β-glucan supplementation group compared with control group.

| Pathway | Different metabolites | The number of different metabolites^a^ | The number of all metabolites^b^ | P value^c^ |
| --- | --- | --- | --- | --- |
| Steroid degradation | Androstenedione, 5-Androstene-3,17-dione, Testosterone, 5alpha-Androstane-3,17-dione, Dehydroepiandrosterone, 3,4-Dihydroxy-9,10-secoandrosta-1,3,5(10)-triene-9,17-dione | 6 | 17 | 0 |
| Biosynthesis of secondary metabolites | Mandelonitrile, Indolin-2-one, Anthranilate, Tropine, Hygrine, Pelletierine, (-)-Hygrine, Trachelanthamidine, 2-Dehydropantoate, (S)-2-Aceto-2-hydroxybutanoate, (R)-3-Hydroxy-3-methyl-2-oxopentanoate, Ecgonine methyl ester, Iridotrial, Pinosylvin, Costunolide, Geranylhydroquinone, Stearidonic acid, Ferruginol, Kaur-16-en-18-al, Neoabietal, Levopimarinal, Abietal, Palustradienal, Isopimaradienal, Kaur-16-en-18-ol, Abietinol, Levopimarinol, Neoabietinol, Taxa-4(20),11(12)-dien-5alpha-ol, Palustradienol, Isopimaradienol, ent-2alpha-Hydroxyisokaurene, ent-11beta-Hydroxycassa-12,15-diene, Abietate, Isopimaric acid, Kaur-16-en-18-oic acid, Levopimaric acid, Neoabietic acid, Palustric acid, 9beta-Pimara-7,15-dien-19-oate, syn-Stemoden-19-oate, Sclareol, Gibberellin A9, Gibberellin A12 aldehyde, 3beta-Hydroxy-9beta-pimara-7,15-diene-19,6beta-olide, 22alpha-Hydroxy-campest-4-en-3-one, (E)-2-Methylpropanal oxime, (Z)-2-Methylpropanal oxime | 48 | 700 | 0.001 |
| Biosynthesis of unsaturated fatty acids | (5Z,8Z,11Z,14Z,17Z)-Icosapentaenoic acid, Icosadienoic acid, 13,16-Docosadienoic acid, Erucic acid | 4 | 20 | 0.012 |
| Aminobenzoate degradation | Anthranilate, 4-Aminobenzoate, (S)-4-Hydroxymandelate, Vanillate, 3-Hydroxy-5-oxohexanoate | 5 | 42 | 0.041 |
| Quorum sensing | N-Hexanoyl-L-homoserine lactone, N-Heptanoylhomoserine lactone, N-Octanoyl-L-homoserine lactone | 3 | 21 | 0.069 |

^a^ The number of differential metabolites that hit the pathway.

^b^ The number of metabolites in the pathway.

^c^ The P value of metabolic pathway enrichment analysis.

**TABLE S3.** Upregulated metabolites negative ion involved pathways in yeast β-glucan supplementation group compared with control group.

| Pathway | Different metabolites | The number of different metabolites^a^ | The number of all metabolites^b^ | P value^c^ |
| --- | --- | --- | --- | --- |
| Ascorbate and aldarate metabolism | Ascorbate, D-Glucuronolactone, L-xylo-Hexulonolactone | 3 | 17 | 0.004 |
| C5-Branched dibasic acid metabolism | Parapyruvate | 1 | 2 | 0.04 |
| Glyoxylate and dicarboxylate metabolism | 2-Hydroxy-3-oxoadipate | 1 | 2 | 0.04 |
| Pentose and glucuronate interconversions | 5-Dehydro-4-deoxy-D-glucuronate, (4S)-4,6-Dihydroxy-2,5-dioxohexanoate | 2 | 17 | 0.045 |

^a^ The number of differential metabolites that hit the pathway.

^b^ The number of metabolites in the pathway.

^c^ The P value of metabolic pathway enrichment analysis.

**TABLE S4.** Upregulated metabolites positive ion involved pathways in yeast β-glucan supplementation group compared with control group.

| Pathway | Different metabolites | The number of different metabolites^a^ | The number of all metabolites^b^ | P value^c^ |
| --- | --- | --- | --- | --- |
| Steroid biosynthesis | Calcidiol, 7-Dehydrocholesterol, Desmosterol, Zymosterol, 5alpha-Cholesta-7,24-dien-3beta-ol, Vitamin D3, Cholesterol, Lathosterol, 5alpha-Cholest-8-en-3beta-ol, 4,4-Dimethyl-5alpha-cholesta-8,14,24-trien-3beta-ol, 4alpha-Methyl-5alpha-ergosta-8,14,24(28)-trien-3beta-ol, 5-Dehydroavenasterol, Campesterol, 24-epi-Campesterol, 4alpha-Methylzymosterol-4-carboxylate | 15 | 34 | 0 |
| Carotenoid biosynthesis | 3'-Hydroxyechinenone, 3-Hydroxyechinenone, Zeaxanthin, Lutein, Rhodopinal, Adonixanthin, Capsorubin, Neoxanthin, Violaxanthin, 9'-cis-Neoxanthin, 9-cis-Violaxanthin, (2S,2'S)-Oscillol, Nostoxanthin, (2'S)-Deoxymyxol 2'-alpha-L-fucoside, Hydroxychlorobactene glucoside | 15 | 55 | 0 |
| Porphyrin and chlorophyll metabolism | Protoporphyrin, Bilirubin, (3Z)-Phytochromobilin, 15,16-Dihydrobiliverdin, 12-Ethyl-8-propyl-3-vinylbacteriochlorophyllide d, D-Urobilinogen, Urobilin, I-Urobilinogen, D-Urobilin, Divinylprotochlorophyllide, 12-Ethyl-8-isobutyl-3-vinylbacteriochlorophyllide d | 11 | 49 | 0 |
| Metabolic pathways | L-Pipecolate, N4-Acetylaminobutanal, L-Lysine, alpha-Isopropylmalate, (2R,3S)-3-Isopropylmalate, (-)-alpha-Bisabolol, Nerolidol, (1E,4S,5E,7R)-Germacra-1(10),5-dien-11-ol, Pregnanediol, Calcidiol, 7alpha-Hydroxycholest-4-en-3-one, 7-Dehydrocholesterol, Desmosterol, Zymosterol, 5alpha-Cholesta-7,24-dien-3beta-ol, Vitamin D3, Cholesterol, Lathosterol, 5alpha-Cholest-8-en-3beta-ol, 4,4-Dimethyl-5alpha-cholesta-8,14,24-trien-3beta-ol, 4alpha-Methyl-5alpha-ergosta-8,14,24(28)-trien-3beta-ol, Campesterol, 5alpha-Campestan-3-one, 24-epi-Campesterol, 7alpha-Hydroxycholesterol, 7alpha-Hydroxy-5beta-cholestan-3-one, 20alpha-Hydroxycholesterol, 22(R)-Hydroxycholesterol, delta-Tocopherol, 2-Methyl-6-phytylquinol, Campestanol, 3alpha,7alpha-Dihydroxy-5beta-cholestane, gamma-Tocopherol, beta-Tocopherol, 6-Oxocampestanol, 2,3-Dimethyl-5-phytylquinol, 6-Deoxocathasterone, 4alpha-Methylzymosterol-4-carboxylate, 3alpha,7alpha,26-Trihydroxy-5beta-cholestane, 3alpha,7alpha,12alpha-Trihydroxy-5beta-cholestane, 3-Dehydroteasterone, Brassinolide, Protoporphyrin, Zeaxanthin, Lutein, Bilirubin, D-Urobilinogen, D-Urobilin, Divinylprotochlorophyllide, Neoxanthin, Violaxanthin, 9'-cis-Neoxanthin, 9-cis-Violaxanthin, Lipid X | 54 | 811 | 0 |
| Sesquiterpenoid and triterpenoid biosynthesis | (2E,6E)-Farnesol, (2Z,6E)-Farnesol, Nerolidol, (1E,4S,5E,7R)-Germacra-1(10),5-dien-11-ol, 8-epi-Cedrol, (3R,6E)-Nerolidol, Patchoulol, (+)-Caryolan-1-ol, Avermitilol, 24-Hydroxy-beta-amyrin | 10 | 63 | 0 |
| Biosynthesis of secondary metabolites | L-Pipecolate, L-Lysine, alpha-Isopropylmalate, (2R,3S)-3-Isopropylmalate, (2E,6E)-Farnesol, (2Z,6E)-Farnesol, (1E,4S,5E,7R)-Germacra-1(10),5-dien-11-ol, 8-epi-Cedrol, alpha-Tocotrienol, 4alpha-Methyl-5alpha-ergosta-8,14,24(28)-trien-3beta-ol, 5-Dehydroavenasterol, Campesterol, Campest-4-en-3beta-ol, 5alpha-Campestan-3-one, delta-Tocopherol, 2-Methyl-6-phytylquinol, Campestanol, gamma-Tocopherol, beta-Tocopherol, 6-Oxocampestanol, 22alpha-Hydroxy-campesterol, 22alpha-Hydroxy-5alpha-campestan-3-one, 2,3-Dimethyl-5-phytylquinol, 6alpha-Hydroxycampestanol, 6-Deoxocathasterone, 3-Dehydroteasterone, Brassinolide, 24-Hydroxy-beta-amyrin, Hopane-29-acetate, Terpendole K, Protoporphyrin, Zeaxanthin, Lutein, Bilirubin, (3Z)-Phytochromobilin, 15,16-Dihydrobiliverdin, 12-Ethyl-8-propyl-3-vinylbacteriochlorophyllide d, D-Urobilinogen, D-Urobilin, Divinylprotochlorophyllide, 12-Ethyl-8-isobutyl-3-vinylbacteriochlorophyllide d, Neoxanthin, Violaxanthin, 9'-cis-Neoxanthin, 9-cis-Violaxanthin | 45 | 700 | 0.001 |
| Lysine degradation | L-Pipecolate, L-Lysine, D-Lysine, (3S)-3,6-Diaminohexanoate, (3S,5S)-3,5-Diaminohexanoate, 2,5-Diaminohexanoate | 6 | 34 | 0.003 |
| Ubiquinone and other terpenoid-quinone biosynthesis | alpha-Tocotrienol, delta-Tocopherol, 2-Methyl-6-phytylquinol, gamma-Tocopherol, beta-Tocopherol, 2,3-Dimethyl-5-phytylquinol | 6 | 53 | 0.026 |

^a^ The number of differential metabolites that hit the pathway.

^b^ The number of metabolites in the pathway.

^c^ The P value of metabolic pathway enrichment analysis.
